# Recognition of Daily Gestures with Wearable Inertial Rings and Bracelets

**DOI:** 10.3390/s16081341

**Published:** 2016-08-22

**Authors:** Alessandra Moschetti, Laura Fiorini, Dario Esposito, Paolo Dario, Filippo Cavallo

**Affiliations:** The BioRobotics Institute, Scuola Superiore Sant’Anna, Viale Rinaldo Piaggio, 34, Pontedera 56025, Italy; l.fiorini@sssup.it (L.F.); d.esposito@sssup.it (D.E.); paolo.dario@sssup.it (P.D.); f.cavallo@sssup.it (F.C.)

**Keywords:** wearable sensors, gesture recognition, activities of daily living, machine learning, sensor fusion

## Abstract

Recognition of activities of daily living plays an important role in monitoring elderly people and helping caregivers in controlling and detecting changes in daily behaviors. Thanks to the miniaturization and low cost of Microelectromechanical systems (MEMs), in particular of Inertial Measurement Units, in recent years body-worn activity recognition has gained popularity. In this context, the proposed work aims to recognize nine different gestures involved in daily activities using hand and wrist wearable sensors. Additionally, the analysis was carried out also considering different combinations of wearable sensors, in order to find the best combination in terms of unobtrusiveness and recognition accuracy. In order to achieve the proposed goals, an extensive experimentation was performed in a realistic environment. Twenty users were asked to perform the selected gestures and then the data were off-line analyzed to extract significant features. In order to corroborate the analysis, the classification problem was treated using two different and commonly used supervised machine learning techniques, namely Decision Tree and Support Vector Machine, analyzing both personal model and Leave-One-Subject-Out cross validation. The results obtained from this analysis show that the proposed system is able to recognize the proposed gestures with an accuracy of 89.01% in the Leave-One-Subject-Out cross validation and are therefore promising for further investigation in real life scenarios.

## 1. Introduction

The population is rapidly ageing worldwide and according to the European Ageing Report [1], by 2060, a large part of the population will be composed of people over 75 years old and the age dependency ratio (ratio of people under 15 and over 65 above people aged between 15 and 65) will increase from 51.4% to 76.6%. Health care systems will be affected by the ageing population due to an increase in the demand for care, especially long-term care, which threatens to decrease the standard of the care process [2]. Indeed, in order to reduce the burden for society, it is important to add healthy years to life, thus decreasing the number of people that will need care. Moreover, in order to reduce long-term care and let older people maintain their independence, new monitoring systems have to be developed [3]. In this way, elderly persons could live longer in their own homes and be monitored both in emergency situations and in daily life [4]. Monitoring people during daily living, apart from recognizing emergency situations, will allow them to maintain a healthy lifestyle (suggesting an increase in physical activity or healthier eating) and, from the caregivers point of view, will allow also a continuous monitoring, which will facilitate to perceive changes in normal behavior and detect early signs of deterioration permitting earlier intervention [5,6]. The ability to recognize the activities of daily living (ADL) is therefore useful to let caregivers monitor the elderly persons. Particularly, among other activities, recognizing eating and drinking activities would also help to check food habits, determining whether people are still able to maintain daily routine and detecting changes in it [7]. Moreover, the opportunity to observe food intake patterns could help to prevent conditions such as obesity and eating disorders, helping individuals to maintain a healthy lifestyle [8,9].

According to Vrigkas et al. [10], activities range from some simple activities, which occur naturally in daily life, like walking or sitting and are relatively easy to recognize, to more complex activities, which may involve the use of tools, and are more difficult to recognize, such as “peeling an apple”. Therefore, research studies intend to find new and efficient ways to identify these kinds of activities. Depending on their complexity, activities can be classified as: (i) gestures, which are primitive movements of the body part that can correspond to a specific action; (ii) atomic actions, which are movements of a person describing a certain motion; (iii) human-to-object and human-to-human interaction, which includes the involvement of two or more persons/objects; (iv) group actions, which are performed by a group of persons; (v) behaviors, which refer to physical actions associated to personality and psychological state; and (vi) events that are high-level activities, which also show social intent. Some of the activities of daily living, like eating or performing acts of personal hygiene, involve movements of the body parts. It is, therefore, possible to recognize gestures to infer the whole activity [11].

The literature suggests that activity recognition is mainly carried out in two ways: with external sensors or with wearable sensors [12]. The former case includes the use of sensors put in the environment or on the objects used by a user to complete the activity. In the latter case, the user wears sensors and the movements made are used to infer activity. Examples of external sensors are cameras and smart homes. In the first case, the recognition of the activities relies on the video analysis of the user [13], which brings into consideration issues like privacy, due to the continuous monitoring of the user, pervasiveness, linked to the necessity for the user to stay in the field of view of the camera, and complexity, linked to the video processing [14]. Smart homes, on the other hand, rely on sensors placed on the objects the person has to interact with, like door switches, motion sensors and RFID tags. This approach requires a large number of sensors to be installed and whenever a new object is added to the system, it has to be tagged and the system needs to be updated. Moreover, if the user does not interact with the supposed object, the system is unable to recognize the activity [15,16].

Thanks to sensors miniaturization and affordability, body worn activity recognition has gained popularity, especially using Inertial Measurement Units (IMUs) and in particular accelerometers. These sensors have demonstrated good potentiality in the recognition of the activities, due to fast response to movement changes [14]. Accelerometers can give high recognition accuracy (up to 97.65% [17]) in activities of daily living such as walking, sitting, lying or transitioning from one to another; however in other kinds of activities, like eating, working at the computer or brushing teeth, the recognition rate decreased [14]. The combination of accelerometers with other sensors like temperature sensors, altimeters or with gyroscopes and magnetometers increases the classification accuracy [18,19]. However it is important in the case of wearable sensors to maintain a low obtrusiveness, so the number of sensors to be worn has to be minimized and they have to not interfere with the activity being performed [14].

In order to recognize gestures involved in ADL, wearable IMU-based sensors can be a low-cost and ubiquitous solution. Thanks to the miniaturization and affordability of these sensors, a lot of existing devices already include accelerometers and gyroscopes, like Smartwatches and Smartphones [17], and can be therefore used to track human movements [20]. Moreover, new devices can be developed to build customized sensors to recognize different kinds of activities trying to be as unobtrusive as possible [21].

The optimal placement of the sensors is still subject of debate. As a matter of fact, sensors can be placed on different parts of human body depending on the activities to be recognized. Common positions to recognize activities such as lying, sitting, walking, running, cycling, and working on a computer are waist, chest, ankle, lower back, thigh and trunk [22]. These placements, however, do not give information about the movements of the arm and of the hand, which are important to identify gestures involving the use of the hand. Adding wrist and finger worn sensors can help obtain this information.

In this context, this work presents an activity recognition system where different gestures usually performed in daily activities are recognized by means of ring and bracelets IMUs placed on the fingers and on the wrist, respectively.

The rest of the paper is organized as follows. Related works are described in Section 2. In Section 3 we described the system used and the methodology together with the experimental setup. Results are described in Section 4 and Section 5 provides a discussion about them. Finally, in Section 6 conclusions and remarks for future works are described.

## 2. Related Works

As stated from the literature, IMUs, and in particular accelerometers, are among the most used sensors for activity recognition [14]. Using these kind of sensors, several supervised machine learning techniques can be used in activity recognition tasks, among these Decision Trees (DT), both C4.5 and CART based [23], and Random Forests (RF), which is a combination of decision trees, are used as classification algorithms [22,24]. Other classification techniques used in gesture and activity recognition problems are K-Nearest Neighbors (KNN) [19,24,25], Naïve Bayes (NB) [17,23,24], Artificial Neural Networks (ANN) [14,23], Support Vector Machines (SVM) [17,26] and Hidden Markov Models (HMM) [16,27]. Often, more than one technique is used to evaluate the recognition ability of the proposed system, in order to compare the performances of the machine learning algorithms.

In recent years, thanks to the advent of Smartwatches and wrist-worn fitness trackers, different studies have recognized different activities with a sensor placed on the wrist , even if in prior studies the use of sensors on the wrist decreases the recognition ability, due to frequent hand movements that add negative information to the activity recognition systems [24].

In particular, Gjoreski et al. [24] compared results obtained with DT, RF, NB, SVM and KNN and found that the accuracy reached a mean value of 72% with the RF technique for activities like walking, standing, sitting, running and other sporting activities. However activities that involve the use of the hands, like eating or drinking, were not taken into account in this work.

In the work of Weiss et al. [28] five different machine learning techniques were applied to recognize activities like walking, jogging and sitting, together with activities like eating pasta, soup, a sandwich, drinking and teeth brushing using a wrist-worn accelerometer. The recognition accuracy was compared to accuracy obtained with the phone accelerometer. In this case, the average accuracy obtained with RF technique in the case of personal model was 93.3%, which dropped to 70.3% when an unknown user was used to test the system previously trained with other users. In particular, the accuracy obtained in the activities that involve the use of the arm reached different values, going from an 84.5% for brushing teeth to 29.0% for recognizing eating a sandwich, which is quite low thinking about real life applications. Indeed, the use of a single sensor in this case did not produce high accuracy in recognizing the different activities.

In order to detect eating and drinking activities, Junker et al. used two wrist inertial sensors coupled with two inertial sensors, one placed on the arm and one on the upper torso [27]. The aim of the study was to detect eating and drinking gestures and a second group of gestures like handshakes, phone up and phone down in a continuous acquisition. Here, using HMM, eating and drinking activities were detected and recognized reaching average recall values of 79% and for the activities of the second group a 93% recall was achieved. However, in this work eating and drinking gestures were not mixed with gestures that involved similar movement of the arm and the hand. Moreover, to perform the different eating gestures, different hands were used, involving therefore different sensors depending on the gestures.

Zhu et al. placed a motion sensor on the hand instead of on the wrist, in order to capture hand gestures like using a mouse, typing on a keyboard, flipping a page, cooking and dining using a spoon [29,30]. Together with these gestures, other activities such as sitting, standing, lying, walking, and transition were recognized. The hand sensor was placed on the ring-finger of the dominant hand and was coupled with sensors placed on the dominant side thigh and on the waist. Moreover, information about the localization in the house was added to the feature using a Vicon motion capture system. The detection and recognition of the gestures reached 100% accuracy for sitting and standing and 80% accuracy for eating. Although recognition rates are good also in this case the eating gestures were compared to gestures that involved different movements of the hand, thereby reducing the possibility of confusing the gestures.

As stated from literature, several studies were conducted on recognizing ADL using wearable sensors [12,19]. Many works are focused on recognizing activities such as lying, sitting, walking, running and other sporting activities and different transitional actions, like sit-to-stand, sit-to-lie, lie-to-stand, etc. [17,22,23,24,31,32,33]. While others include more complex gestures, like using the mouse, typing on a keyboard, eating, drinking, teeth brushing, etc., however, they do not focus on similar ones. Indeed, these kinds of gesture are grouped putting together gestures that are different to the others, so that the probability of confusing them is lower [25,28,29,30].

Therefore this work aims to go beyond the state of the art by proposing a system where the ability of finger- and wrist-worn sensors are investigated to recognize similar gestures that are part of daily living, such as eating, drinking and some personal hygiene. The purpose of this work is therefore to find the best combination of sensors, in terms of the trade-off between recognition ability and obtrusiveness of the system, in order to distinguish among similar gestures, which involve the use of the hand that is moved to the mouth/head. The recognition problem will be analyzed using two different supervised machine learning algorithms and will be evaluated in a personal analysis (user used for training and testing the machine learning algorithm), to evaluate the intrapersonal variability of the gestures, and then it will be generalized considering the case where the test user is not part of the training set, so that the system can be generalized to new users without the need for further training.

## 3. Materials and Methods 

In this section, we describe the methodology applied in this study, including the sensors used, data acquisition and processing, the classifiers used, the analysis carried out, and the evaluation of the performance.

### 3.1. System Description

Data were collected using IMUs, in particular, an evolution of the SensHand wearable device [34] and a wrist sensor unit. The SensHand is a wearable device developed using inertial sensors integrated into four INEMO-M1 boards with dedicated STM32F103xE family microcontrollers (ARM 32-bit Cortex™-M3 CPU, STMicroelectronics, Milan, Italy). Each module includes LSM303DLHC (6-axis geomagnetic module, dynamically user-selectable full scale acceleration range of ±2 g/±4 g/±8 g/±16 g and a magnetic field full-scale of ±1.3/±1.9/±2.5/±4.0/±4.7/±5.6/±8.1 gauss, finally set on ±8 g and ±4.7 gauss respectively, STMicroelectronics, Milan, Italy) and L3G4200D (3-axis digital gyroscope, user-selectable angular rate full-scale of ±250/±500/±2000 deg/s, finally set on ±2000 deg/s, STMicroelectronics, Milan, Italy) and I2C digital output. Both the coordination of the modules and synchronization of the data are implemented through the Controller Area Network (CAN-bus) standard. The coordinator module collects data at 50 Hz and transmits them through the Bluetooth V3.0 communication protocol by means of the SPBT2632C1A (STMicroelectronics, Milan, Italy) Class 1 module. Data were collected on a PC by means of custom interfaces developed in C# language. A small, rechargeable and light Li-Ion battery, integrated into the coordinator module, supplies power to the system. The SensHand sends the data collected from accelerometers and gyroscopes, and the Euler Angles (Roll, Pitch and Yaw) that describe the orientation of each module in the 3D space and that are evaluated as in [11] from the quaternions obtained through an embedded quaternion-based Kalman filter, adapted from [35]. 

The wrist sensor was developed using the same technologies as SensHand and has analogue features including the iNEMO-M1 inertial module. Data are collected and transmitted via Bluetooth serial device at 50 Hz. Even in this case, the device has a small battery that supplies the system. The wrist sensor sends data coming from the magnetometer, in addition to the data of the accelerometer, the gyroscope and the Euler Angles.

The data of the accelerometers and gyroscopes are filtered on board using a fourth-order low pass digital filter with a cutoff frequency of 5 Hz, in order to remove high frequency noise and tremor frequency bands [34]. The sensors were adequately calibrated both in static and dynamic conditions to calculate the offset and sensitivity that affect measurements. 

**Figure 1:**
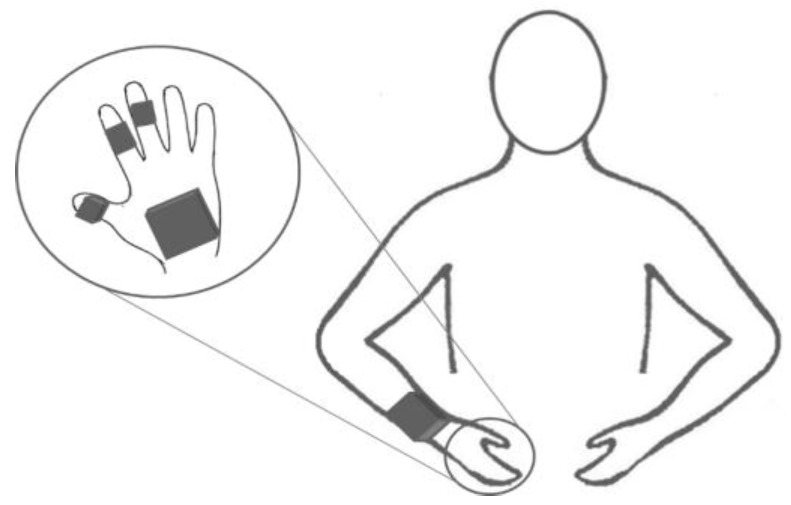
Placement of inertial sensors on the dominant hand and on the wrist. In the circle a focus on the placement of the SensHand is represented, while in the half-body figure the position of the wrist sensor is shown.

The position of the sensors was chosen according to literature [24,27,28], but also by analyzing the kind of gestures that need to be identified. The wrist is a good location for wearable sensors, due to the low obtrusiveness, as demonstrated by commercial devices as Smartwatches. However the proposed gestures to be studied are similar and involve the use of the hand and the grasping of different objects. Therefore, in addition to the wrist-worn device we added sensors to the hand to analyze whether having information from the fingers mostly involved in the grasping of the objects [36] could improve the recognition rate. The described devices were therefore put on the dominant hand and on the wrist as depicted in Figure 1. In particular, the coordinator of SensHand was placed on the backside of the hand, while the three modules were placed on the distal phalange of the thumb, the intermediate phalange of the index and middle fingers. The second sensor device was placed on the wrist, like a Smartwatch.

### 3.2. Data Collection and Experimental Setup

**Figure 2:**
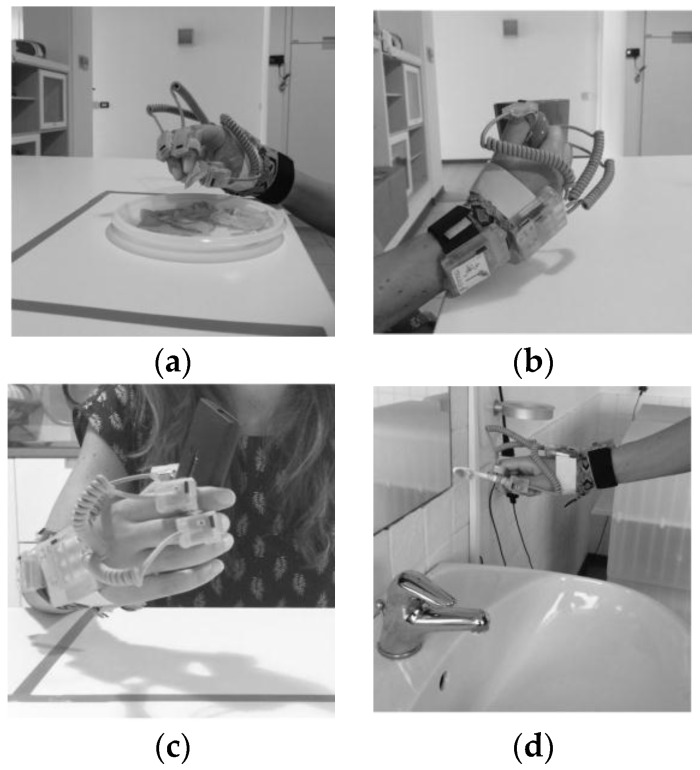
Focus on grasping of objects involved in the different gestures (**a**) grasp some chips with the hand (HA); (**b**) take the cup (CP); (**c**) grasp the phone (PH); (**d**) take the toothbrush (TB).

**Figure 3:**
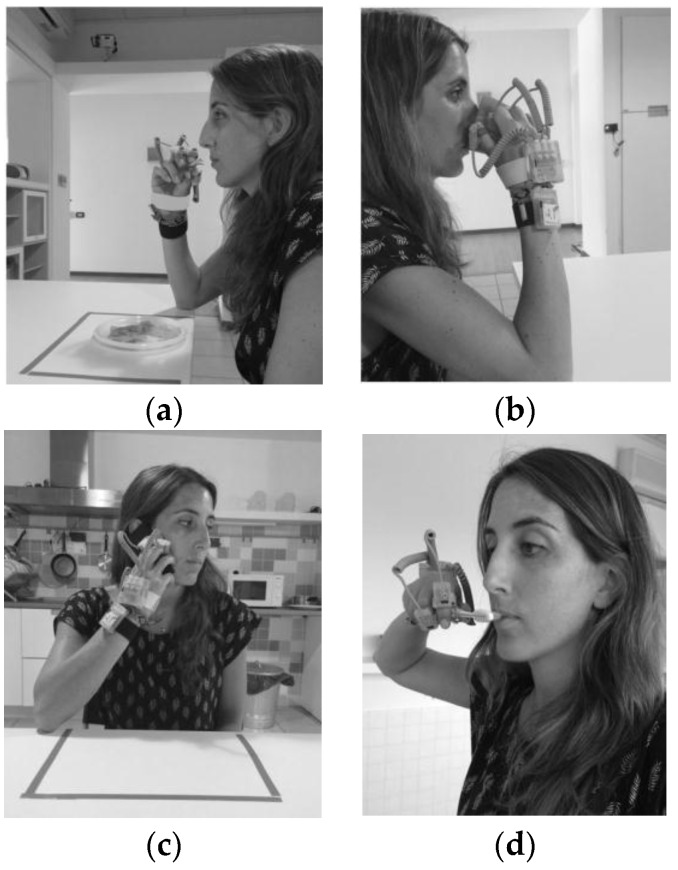
Example of (**a**) eating with the hand gesture (HA); (**b**) drink from the cup (CP); (**c**) answer the telephone (PH); (**d**) brushing the teeth (TB).

Nine gestures were selected that can be used to recognize some of the ADL performed by a person at home. These gestures were also chosen because they are similar to each other and involve the use of the hand moving to the head/mouth. Moreover, we wanted to recognize different eating and drinking gestures, partly according to the state of the art [16,27] and partly to obtain information that could be used in future to monitor food intake. Some examples of these gestures are reported in Figure 2, where a focus on the grasping of different objects involved in the gestures is shown, and in Figure 3. The chosen gestures were:

1. Eat with the hand (HA). In this gesture the hand was moved to the mouth and back to the table (Figure 2a and Figure 3a).
2. Drink from a glass (GL). Persons were asked to grasp the glass, move it to the mouth and back to the table.
3. Eat some cut fruit with a fork from a dish (FK). Participants had to take a piece of fruit with the fork and eat it and back to the table holding the fork in the hand, without leaving it.
4. Eat some yogurt with a spoon from a bowl (SP). Users had to use the spoon, load it and move it to the mouth and back to the table holding it in the hand.
5. Drink from a mug (CP). Persons were asked to grasp the mug, move it to the mouth and then leave it on the table (Figure 2b and Figure 3b).
6. Answer the telephone (PH). Persons were asked to take the phone from the table, move it to the head and back on the table after a few seconds (Figure 2c and Figure 3c).
7. Brush the teeth with a toothbrush (TB). The gesture consisted of taking the toothbrush from the sink, move it to the mouth to brush the teeth and move it back to the sink (Figure 2d and Figure 3d).
8. Brush hair with a hairbrush (HB). In this gesture the hairbrush was taken from the sink, moved to the head, used two/three times and then back to the sink.
9. Use a hair dryer (HD). Users had to take the hair dryer from the sink, move to the head to dry their hair and move it back to the sink.

In order to evaluate whether the proposed system of sensors is able to recognize the chosen gestures, twenty young healthy participants (11 females and 9 males, whose ages ranged from 21 to 34 (29.3 ± 3.4)) were involved in the experimental session. The experiments were carried out in Peccioli, Pisa, in the DomoCasa Lab, a 200 m^2^ fully furnished apartment [37]. This location was chosen to make the participants perform the gestures in a realistic environment, avoiding unnatural movements linked to a laboratory setting as much as possible.

The testers, after having signed an informed consent form, were asked to make all the described gestures without any constriction of the way in which movements were performed or objects were taken. Each gesture was performed 40 times continuously and manually labeled to identify the beginning and the end of each of the 40 gestures. At the beginning of each sequence, participants were asked to stay still with the hand and the forearm lying on a plane surface to permit a static acquisition in order to calibrate each session and compare the position of the sensors among the different gestures of the session, referring to the first gesture made (eating with the hand). At the end of the experimental session, users were asked if the system had influenced the movements. Our intention was not to perform a usability and acceptability study, but to know if they could perform the gestures in a natural way. The users answered that the worn sensors did not influence the way in which the gestures were performed.

### 3.3. Signal Processing and Analysis/Classification

The first step in the data processing pipeline was therefore the synchronization of the signals coming from the two devices and the segmentation of the signal into single gestures according to the labels. Features are generated from each gesture.

By analyzing acceleration signals, time domain features (like mean, standard deviation, variance, mean absolute deviation (MAD)) and frequency domain features (like Fourier Transform and Discrete Cosine Transform) can be extracted [14,26]. In some cases, roll and pitch angles of the sensors are added to the features in order to include information about the orientation of the body segment where the sensor is placed [27].

According to the literature [14,27] and from the analysis of the raw data, for each gesture the 3-axis acceleration and the roll and pitch angle for each module were considered. In particular, the following features were evaluated for each labeled gesture:

- Mean, standard deviation, root mean square and MAD of the acceleration;
- Mean, standard deviation, MAD and minimum and maximum of the roll and pitch angle.

When considering all the sensors the dataset included 7200 gestures with 110 features ((12_accelerometers_ + 10_angle_) × 5) labeled with the corresponding gesture. This dataset is used by the machine learning algorithm to build a recognition model in the training phase and to recognize the gestures in the classification phase.

Before using the dataset for the classification algorithm, the matrix of the features was normalized to avoid distortion due to data heterogeneity. A Z-norm was computed to have zero mean and a unit standard deviation.

In this work we compared two different classification algorithms commonly used in gesture and activity recognition problems [14,23,25] to recognize the gestures and have a quantitative analysis of the best combination of sensors:

- **Decision Tree (DT):** These are models built on test questions and conditions. Nodes of the tree represent tests, branches represent outcomes and the leaf nodes are the classification labels. Each branch from the root to the leaf node is a classification rule. DTs are widely used in activity recognition problems. Here, we used a built-in function of MATLAB which implements a CART (Classification and Regression Trees) decision tree [14].
- **Support Vector Machine (SVM):** this classification technique was developed for binary classification where the aim was to find the Optimal Separating Hyperplane between two datasets. SVM cannot deal with a multiclass problem directly, but usually solves such problems by decomposing the problem into several two-class problems [26,38]. The strategy adopted in this work was a One-versus-One strategy. A third-order polynomial function for the kernel was chosen and the number of iterations was set at 10^6^. To implement the SVM we used the function implemented by Cody Neuburger [39] that is an adaptation of the code of multiSVM function developed by Anand Mishra [40]

In order to achieve the proposed goals, starting from the complete dataset (110 features) different analyses were carried out in order to evaluate the classification performance by changing the combination of sensors. Particularly, we considered the full system as the gold standard and our interest was focused on the evaluation of the use of the wrist device, of the index sensor and some combination of them with the thumb sensor (as summarized in Table 1). 

To evaluate the recognition of the gesture two approaches were used: a personal approach and a leave-one-out approach. In the personal one the training set and the test set come from the same user, while in the leave-one-subject-out (LOSO) cross validation technique, the training dataset is made by all the users except the one who is going to be used as a test dataset [15].

As regard the personal model, a personal dataset for each participant was created and permuted to mix the nine gestures, then a 60/40 training/test partition of the matrix was made. In order to compare the results coming from the different users, the same permutation indices were used to permute the personal dataset matrix.

In LOSO technique, the training dataset was built on 19 users and the remaining user was used to test the model and evaluate the classification. This analysis was performed leaving out each of the twenty users. 

We evaluated the proposed sensors’ configurations to recognize the nine gestures by means of personal and LOSO approaches. In both analyses, for each configuration and each classification algorithm the confusion matrix was created in order to evaluate the accuracy, the precision, the recall, the F-measure and specificity that are used to summarize the performances of the classification algorithms. Defining True Positives (TP) as the number of correct classifications of positive instances, True Negatives (TN) as the number of correct classifications of negative instances, False Positives (FP) and False Negatives (FN) as the numbers of negative instances classified as positive and the number of positive instances classifies as negative respectively, accuracy, precision, recall, F-measure and specificity can be described as [14,22]:

(1) $$\text{ACCURACY}=\frac{\text{TP}+\text{TN}}{\text{TP}+\text{TN}+\text{FP}+\text{FN}}$$

(2) $$\text{PRECISION}=\frac{\text{TP}}{\text{TP}+\text{FP}}$$

(3) $$\text{RECALL}=\frac{\text{TP}}{\text{TP}+\text{FN}}$$

(4) $$F-\text{measure}=2\times \frac{\text{Precision}\times \text{Recall}}{\text{Precision}+\text{Recall}}$$

(5) $$\text{SPECIFICITY}=\frac{\text{TN}}{\text{TN}+\text{FP}}$$

## 4. Results

DT and SVM F-measure and accuracy results for personal analysis of the different sensors’ configurations are reported in Table 2. These results were obtained by averaging the results obtained for each participant.

Both the considered classifiers have reached accuracy results higher than 94%, in all the configurations analyzed, although the F-measure and accuracy obtained with the SVM outperform the ones obtained with the DT. Indeed, the F-measure and accuracy rate evaluated for the DT are around 95%, while for the SVM they are around 99%.

**Figure 4:**
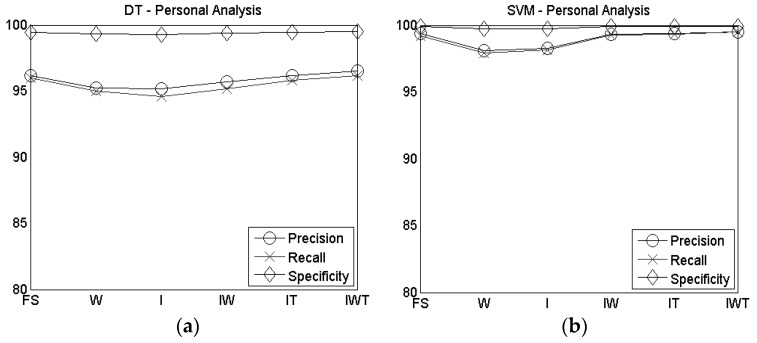
Precision, recall and specificity of personal analysis for (**a**) DT and (**b**) SVM.

In Figure 4 the personal analysis results of the precision, recall and specificity are reported for DT (Figure 4a) and SVM (Figure 4b). Even in this case, the SVM outperforms the DT, although the values obtained with the DT are still high (>94%).

In general, except for the specificity where results are higher than 99% for all the proposed configurations, by increasing the number of considered sensors results are improved for both DT and SVM, although the use of the combined index, wrist and thumb sensors brought to a slightly higher accuracy and F-measure than the full system configuration, as shown in Table 2.

Table 3 shows the F-measure and accuracy results for the LOSO approach analyzed using the DT and the SVM. The values are the average of the results for each participant, reaching 89.06% accuracy for the FS configuration with DT and 91.79% with SVM, and an F-measure of 88.05% for the FS with DT and 91.14% with SVM. Both accuracy and F-measure decrease significantly in the recognition of the gestures in the case of a single sensor being used respective to the FS configuration (in the W configuration around 20% for DT and more than 25% for SVM, while in the I configuration around 7% for DT and more than 9% for SVM) and in W configuration the results obtained with the DT are slightly higher than the ones obtained with the SVM. 

As shown in Table 3, when using the DT for classification, both accuracy and F-measure increase when more sensors are considered, reaching the highest value for the full system configuration (accuracy is 89.06% and F-measure is 88.05%). Alternatively, with the SVM the accuracy and the F-measure obtained with the full system and the ITW configuration are similar (91.79% and 91.32%, respectively for the accuracy and 91.14% and 90.84%, respectively for the F-measure). The lowest accuracy is obtained for the wrist sensor configuration both for DT (68.85%) and SVM (65.03%), while the index sensor configuration improves the accuracy in the recognition (82.07% with DT and 82.04% with SVM), even if the coupling of two sensors performs better. As shown in Table 3, the F-measure for the wrist sensor alone is the lowest both for DT (66.95%) and SVM (62.26%), but this increases when the index sensor configuration is considered instead (80.95% with DT and 81.19% with SVM).

**Figure 5:**
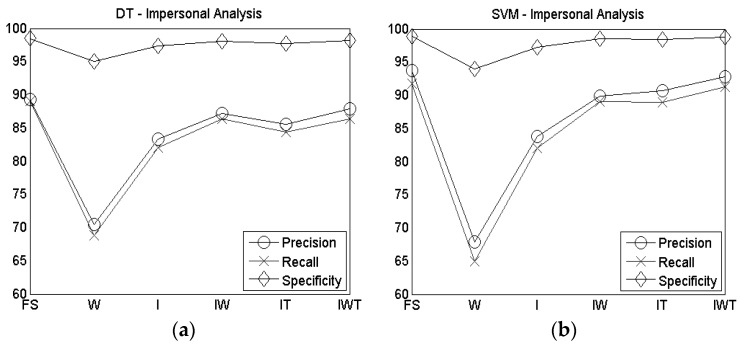
Precision, recall and specificity of impersonal analysis for (**a**) DT and (**b**) SVM.

The use of the wrist sensor also produces moderate results in terms of precision, recall and specificity, both for DT and for SVM as shown in Figure 5. Both DT and SVM produce high accuracy (86.62% and 91.32% respectively) and F-measure (85.26% and 90.84% respectively) when index, wrist and thumb sensors were considered (Table 3), and even in the IW configuration high accuracy and F-measure were reached (86.33% for DT and 89.01% for SVM for the accuracy and 85.47% for DT and 88.40% for SVM for the F-measure).

**Figure 6:**
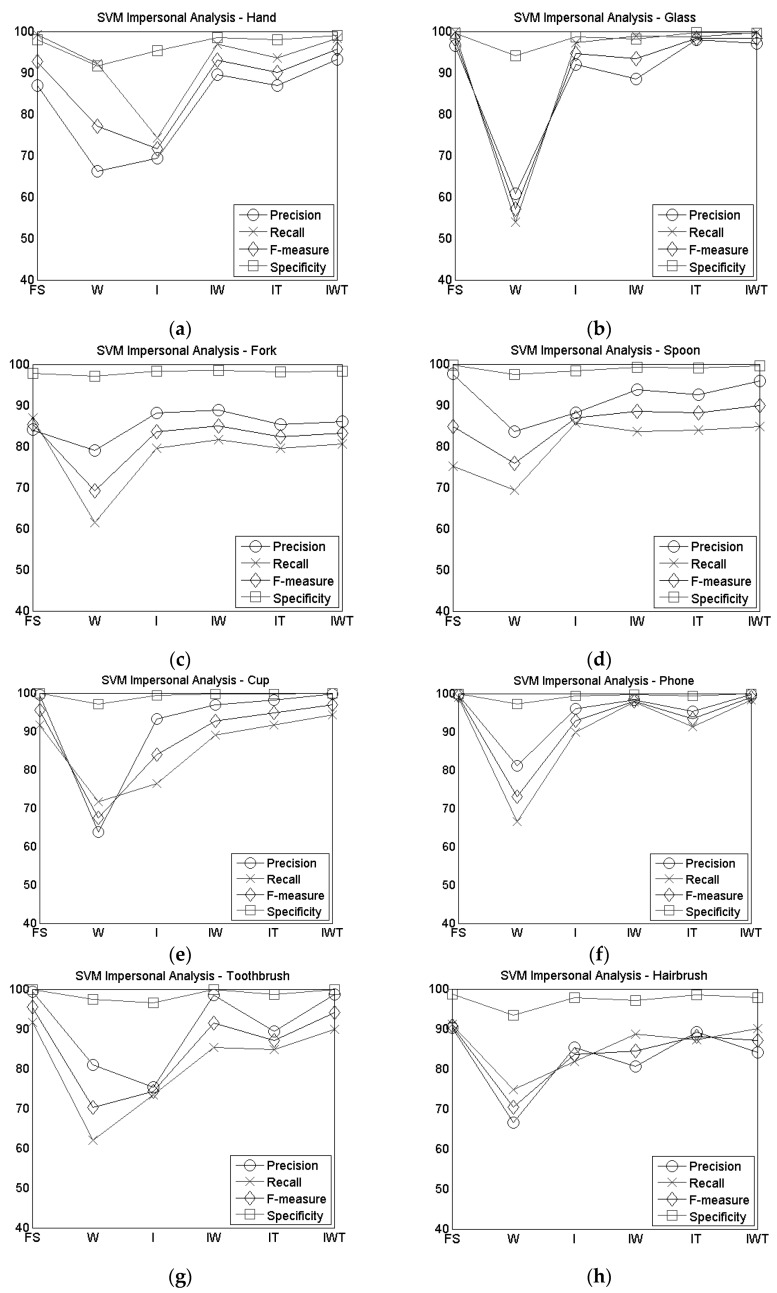

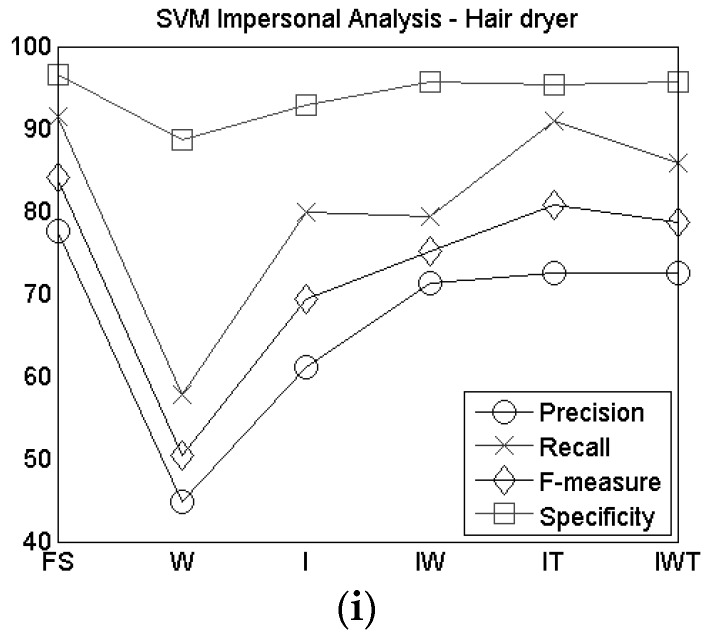
Precision, recall and specificity of SVM impersonal analysis for (**a**) Hand gesture; (**b**) Glass gesture; (**c**) Fork gesture; (**d**) Spoon gesture; (**e**) Cup gesture; **(f**) Phone gesture; (**g**) Toothbrush gesture; (**h**) Hairbrush gesture; (**i**) Hair dryer gesture.

Focusing on the LOSO technique, except for the wrist sensor configuration, where DT gave better results than SVM, and for the index sensor configuration, where the performance of DT and SVM are similar, in all other cases, SVM outperforms DT. For this reason, Figure 6 shows the precision, recall, F-measure and specificity for each gesture achieved with the SVM technique when changing the combination of sensors used. The values of precision, recall, F-measure and specificity of all the configurations, except the IW one, are reported from Table A1 to Table A5 in Appendix A.

Precision, recall, F-measure and specificity gave different results according to the different combination of sensors, however, considering the F-Measure, the hair dryer gesture achieved the worst value in all configurations.

Among the different combinations of sensors, we focused our attention on the IW one, due to our interest in finding the best combination that achieved a good recognition rate while maintaining low obtrusiveness. In particular, in the LOSO technique, the SVM outperforms the DT in terms of accuracy (89.01%), F-measure (88.40%) (Table 3), precision (89.93%) and recall (89.01%) (Figure 5). The values of precision, recall, F-measure and specificity for each gesture achieved with the SVM technique for the IW combination of sensors are shown in Table 4. The F-measure shows that the hardest gesture to recognize is the use of the hair dryer, while taking the phone from the table reached a high value of F-measure (98.06%). Both drinking from the cup and from the glass reached quite high values of F-measure (93.44% and 92.77%, respectively). Among the eating gestures, eating with the hand showed the highest value of F-measure (93.09%), while the gestures involving fork and spoon presented slightly lower values, although they were still good (85.08% and 88.43% respectively).

## 5. Discussion

Six different combinations of inertial sensors were tested to recognize nine gestures that are part of daily activities. These were a full system (sensors on thumb, index and middle finger, on the backside of the hand and on the wrist), a sensor on the wrist, a sensor on the index finger, sensors on the index and wrist, sensors on the index finger and thumb and sensors on the index, thumb and wrist (see Table 1). Two machine learning algorithms, DT and SVM, were adopted for the classification problem. The aim of this work was to evaluate the ability to distinguish similar gestures that are involved in the ADL and that require hand/arm motions around the head and the mouth. In effect, the proposed protocol included, beyond personal hygiene, different eating (by mean of the hand, using a fork and using a spoon) and drinking activities (with a glass and with a cup). This information could be used in future applications to monitor the food intake patterns, which are important for people of all ages, especially for elderly persons, to avoid eating disorders and obesity and prevent related diseases [4,8,9].

In the case of personal analysis, all the sensor combinations produced high values of accuracy and F-measure in the recognition of the different gestures, both for the DT and SVM algorithm (see Table 2). In this case, classifiers are trained on the gestures made by a single user and then tested on gestures made by the same user. This kind of study was useful to evaluate the intrapersonal variability in performing the gestures. According to the obtained results, persons perform the same gesture in a very similar way all the times they have to.

Using the LOSO technique, in general the SVM algorithm produced better results compared to DT, except for the case of the use of the wrist sensor alone. When using the single sensor on the wrist, the accuracy obtained is moderate (68.85% in DT and 65.03% in SVM, see Table 3) as for the F-measure (66.95% in DT and 62.23% in SVM see Table 3). This could be due to the fact that an important difference among the considered gestures is the way in which the objects are taken and handled, so the wrist sensor alone cannot distinguish accurately. For example the drinking gestures seen from the point of view of the wrist are more or less the same gesture, what differs is the grasping of the used object. As a matter of fact, similar gestures, like drinking from a cup and from the glass (F-measure equal to 57.12% and 67.49% respectively, as shown in Table A2 in Appendix A) are often wrongly classified, the same as the hair dryer and the hairbrush gestures (F-measure equal to 50.49% and 70.48% respectively, as shown in Table A2 in Appendix A). Regarding these gestures, the F-measure, precision, recall and specificity increase when more information about the finger is given, as it can be seen in Figure 6b,e,h,i, comparing the W and the I configurations. The results obtained regarding the recognition of eating gestures with the wrist sensors are quite similar to some studies found in the state of the art [5] or lower with respect to others [25], where the eating activities were mixed with activities, like walking, lying and standing. 

The accuracy and the F-measure increase by about 14% in both algorithms when the index sensor is used. Even if the recognition rate is not so high, when using the index sensor with the SVM classification algorithm (Table 3), the system is more able to recognize the different gestures. The index sensor, especially thanks to the information regarding the orientation of the finger, provides knowledge about the grasping of the object and therefore is able to distinguish different gestures and even similar gestures are more often properly classified. For example, as shown in Figure 6b,e (and in Table A3 in Appendix A), the F-measure of the cup and the glass rises to 83.99% and to 94.59% respectively and the hairbrush and the hair dryer up to 83.55% and 69.30% respectively, compared to the wrist configuration. However, even if using the index finger sensor instead of the wrist one increases the performances, analyzing the results of the use of a single sensor it can be seen that this is still not sufficient to recognize these gestures accurately and different gestures are often confused.

The combination of two sensors improves the accuracy of the recognition, especially when using the SVM algorithm, as it can be seen from Figure 6. The use of the thumb and index sensors together increases the accuracy value up to 88.89% and the F-measure up to 88.32% (Table 3). The thumb increases the knowledge given about the object that is handled. Similar gestures such as drinking from the cup and drinking from the glass are less often confused with respect to a single sensor configuration, obtaining higher values of precision ( 98.01% and 98.13% and a F-measure of 98.38% and 94.76%, respectively) as shown in Figure 6b,e (and in Table A4 in Appendix A). On the other hand, using this configuration of sensors, eating with the spoon and eating with the fork are often mutually confused, due to the fact that participants usually hold the two utensils in the same way. As shown in Figure 6c,d, the precision in the recognition of these two gestures increases when the two sensors considered are the index finger and the wrist. In particular, as shown in Table 4, the precision in the recognition of the fork is 88.84%, while the one of the spoon is 93.83%. By combining these sensors we can obtain information both about the grasping and the movement made by the wrist, thus increasing the F-measure in the recognition of some gestures, such as eating with the hand, taking the phone from the table and using the toothbrush compared to the IT combination, as shown in Figure 6a,f,g respectively. When considering the overall accuracy and F-measure, the IW configuration presents similar results to the IT configuration (89.01% accuracy and 88.40% F-measure, as shown in Table 3).

In order to combine more information about the grasping of the objects, together with the knowledge about the movement given by the wrist, a combination of three sensors (thumb, index and wrist) was tested. In this case, the accuracy increased up to 91.32% (Table 3) which is comparable to the full system (91.79%), showing that the information from the middle finger and the backside of the hand are not strictly necessary to improve the gesture recognition. In the three-sensors combination, classification of similar gestures is improved, reaching F-measure values of 98.40% and 96.98% for the glass and the cup gestures, respectively, as shown in Figure 6b,e (and in Table A5 in Appendix A). The F-measure in the recognition of the nine gestures improves for almost all the gestures with respect to the two-sensors configurations, except for the fork gesture, which is better recognized by the IW combination, as shown in Figure 6c, and the hairbrush (HB) and hair dryer (HD) which are better recognized by the IT combination of sensors, as shown in Figure 6h,i. The lower recognition rates of these two gestures (HB, HD) can be explained by the similarity of the movements of the gestures made by the participants that is overcome when using only the thumb and the index finger sensors as they provide more focused information about the movement and orientation of the two fingers. On the other hand, the fact that the F-measure of the eating with the fork gesture is higher with the IW configuration (Figure 6c) can be explained by the fact that some of the participants held the fork in a different way with respect to the majority of the users, so increasing the knowledge about the grasping could induce wrong classification. Therefore, in these cases, the combination of three sensors does not improve the recognition of the gestures, due also to the fact that especially for the hair dryer and the hairbrush the wrist performances are very low, thus influencing the total recognition rate in case it is added to the index finger and the thumb sensors.

Considering the performances of the different combination of sensors in recognizing the proposed gestures, it is noticed that the best performance is obtained with the IWT combination. Despite the high accuracy values reached with this configuration, the use of the three sensors can be cumbersome for the user. In addition to the sensor on the wrist, people should wear two sensors on the hand (index and thumb), as, even if in the proposed gestures they are not influencing the movements, in daily life they can be obtrusive and interfere with other gestures made by the users. Moreover, the increase in the performance from a two-sensors configurations (IT and IW) to a three-sensors one (IWT) is not as meaningful as the increase from one-sensor configurations (W and I) to two-sensor configurations (IT and IW), so it does not justify the use of a third sensor.

Even if the use of two sensors (IT and IW combination) gave slightly lower accuracy values with respect to the IWT combination (as shown in Figure 6), they can be less invasive resulting in a good trade-off between performance and obtrusiveness. In particular, as shown in Figure 6 comparing IW and IT, the obtained performances are quite similar (see also Table 3), but considering that the wrist sensor is already used for different activity recognition tasks [17,24], due to its unobtrusiveness, the use of the wrist sensor instead of the thumb one seems to be a good choice. As a matter of fact, the wrist sensor has already been used and, as stated from previous works [17,23], it allows to recognize different activities, like standing, walking, running, sit-to-stand and others, showing good results (F-measure >90% [17] and recognition rate >89% [23]). Therefore, adding a ring like sensor on the intermediate phalange of the index finger to the wrist sensor would facilitate recognizing the gestures proposed in this work, beyond other kinds of activities, maintaining a low obtrusiveness.

## 6. Conclusions

The aim of this work was to investigate how different IMUs could recognize similar gestures and to determine the best configuration of sensors in terms of performance. The IW combination shows good results in term of recognition accuracy and F-measure even in the LOSO cross validation technique. The results obtained with the IW combination showed good capability to recognize different gestures, even when a new user is tested on a model trained by different users, while still maintaining low obtrusiveness. The obtained results are therefore promising to further investigate the use of these wearable sensors in a real life scenario. In the future we plan to conduct more studies and data acquisition in order to identify and recognize the same gestures in a more real context, like eating a full meal, and in a continuous acquisition by mixing them with spurious movements and other non-classified gestures. Different methods will also be tested in order to make the system able to recognize the beginning of a movement and then, using a sliding window approach, to finally identify the gesture (getting inspiration from the work of [27]). Different machine learning algorithms will be also analyzed to compare the results obtained and find the best solution for final online recognition. Moreover, the sensor system and the recognition model should also be applied in long-term analysis, in order to determine the ability to identify and recognize the proposed gestures.

To improve the analysis of the IW combination, future acquisition could be done with a ring like device for the index phalange, like the one presented in [41] and a custom device for the wrist, which will be more wearable. Usability and acceptability studies could be performed with this configuration of sensors, to analyze the comfort and obtrusiveness of the sensors.

Finally, in order to increase the accuracy of the recognition of gestures, information about the localization of the user in the house (kitchen or bathroom for the considered gestures) can be added to the features to be taken into account. In this way, it could be possible to better distinguish between similar gestures that are performed in different rooms, for example, the use of the hair dryer, which was the least-well recognized gesture in all the sensor configurations.

## Figures and Tables

**Table 1:** Combination of sensors used for the analysis for each model.

Combination of Sensors					
Full system (FS)	Wrist (W)	Index Finger (I)	Index finger + Wrist (IW)	Index finger + Thumb (IT)	Index finger + Wrist + Thumb (IWT)

**Table 2:** Average F-measure (%) and accuracy (%) of personal analysis.

	F-Measure		Accuracy	
	DT	SVM	DT	SVM
Configuration	Mean ± SD	Mean ± SD	Mean ± SD	Mean ± SD
FS	95.91 ± 2.37	99.29 ± 1.27	95.83 ± 2.44	99.34 ± 1.20
W	94.90 ± 2.76	97.93 ± 1.76	94.93 ± 2.63	97.99 ± 1.73
I	94.55 ± 3.27	98.09 ± 1.37	94.62 ± 3.19	98.13 ± 1.41
IW	95.19 ± 2.03	99.26 ± 0.86	95.24 ± 2.01	99.31 ± 0.84
IT	95.79 ± 2.17	99.34 ± 0.76	95.76 ± 2.17	99.38 ± 0.74
IWT	96. 16 ± 2.04	99.48 ± 0.76	96.22 ± 2.00	99.51 ± 0.72

**Table 3:** Average F-measure (%) and accuracy (%) of LOSO analysis with standard deviation (SD).

	F-measure		Accuracy	
	DT	SVM	DT	SVM
Configuration	Mean ± SD	Mean ± SD	Mean ± SD	Mean ± SD
FS	88.05 ± 7.71	91.14 ± 8.11	89.06 ± 6.58	91.79 ± 9.86
W	66.95 ± 11.95	62.23 ± 14.04	68.85 ± 10.75	65.03 ± 12.03
I	80.95 ± 9.07	81.19 ± 11.10	82.07 ± 8.08	82.04 ± 8.07
IW	85.47 ± 8.07	88.40 ± 7.47	86.33 ± 7.19	89.01 ± 9.10
IT	83.54 ± 7.50	88.32 ± 8.44	84.35 ± 6.82	88.89 ± 8.23
IWT	85.26 ± 7.95	90.84 ± 7.75	86.62 ± 6.71	91.32 ± 6.09

**Table 4:** Values of precision, recall, F-Measure, specificity of LOSO model SVM IW (all values are expressed in %). The gestures are classified as follows: HA stands for Hand, GL stands for Glass, FK stands for Fork, SP stands for Spoon, CP stands for Cup, PH stands for Phone, TB stands for Toothbrush, HB stands for Hairbrush and HD stands for Hair dryer.

	HA	GL	FK	SP	CP	PH	TB	HB	HD
Precision	89.60	88.58	88.84	93.83	96.87	98.36	98.56	80.57	71.27
Recall	96.88	98.88	81.63	83.63	89.00	97.75	85.38	88.63	79.38
*F*-Measure	93.09	93.44	85.08	88.43	92.77	98.06	91.49	84.40	75.10
Specificity	98.43	98.22	98.60	99.24	99.65	99.77	99.83	97.09	95.75

## Appendix A

Table A1, Table A2, Table A3, Table A4 and Table A5 show the precision, recall, F-measure and specificity for each gesture achieved with the SVM technique. The tables presents results obtained for the Full System(FS), the Wrist sensor (W), the Index sensor (I), the Index and Thumb sensors (IT) and the Index, Wrist and Thumb sensors (IWT). The gestures are classified as follows: HA stands for Hand, GL stands for Glass, FK stands for Fork, SP stands for Spoon, CP stands for Cup, PH stands for Phone, TB stands for Toothbrush, HB stands for Hairbrush and HD stands for Hair dryer.

**Table A1:** Values of precision, recall, F-Measure, specificity of LOSO model SVM FS (all values are expressed in %).

	HA	GL	FK	SP	CP	PH	TB	HB	HD
Precision	86.97	96.61	84.04	97.56	99.59	99.75	99.46	90.33	77.71
Recall	99.25	99.88	86.88	75.13	91.63	99.00	91.75	91.13	91.50
F-Measure	92.70	98.22	85.43	84.89	95.44	99.37	95.45	90.73	84.04
Specificity	97.99	99.52	97.82	99.75	99.95	99.97	99.93	98.69	96.55

**Table A2:** Values of precision, recall, F-Measure, specificity of LOSO model SVM W (all values are expressed in %).

	HA	GL	FK	SP	CP	PH	TB	HB	HD
Precision	66.28	60.79	78.97	83.58	63.81	81.22	81.05	66.67	44.85
Recall	92.13	53.88	61.50	69.38	71.63	66.50	62.00	74.75	57.75
F-Measure	77.09	57.12	69.15	75.82	67.49	73.13	70.25	70.48	50.49
Specificity	91.69	94.11	97.10	97.54	97.09	97.25	97.42	93.47	88.60

**Table A3:** Values of precision, recall, F-Measure, specificity of LOSO model SVM I (all values are expressed in %).

	HA	GL	FK	SP	CP	PH	TB	HB	HD
Precision	69.39	92.07	88.11	88.17	93.28	95.99	75.35	85.29	61.13
Recall	74.25	97.25	79.63	85.75	76.38	89.88	73.38	81.88	80.00
F-Measure	71.74	94.59	83.65	86.95	83.99	92.83	74.35	83.55	69.30
Specificity	95.30	98.71	98.39	98.27	99.31	99.43	96.52	97.89	92.83

**Table A4:** Values of precision, recall, F-Measure, specificity of LOSO model SVM IT (all values are expressed in %).

	HA	GL	FK	SP	CP	PH	TB	HB	HD
Precision	86.89	98.01	85.27	92.56	98.13	95.43	89.34	89.26	72.51
Recall	93.63	98.75	79.63	84.00	91.63	91.38	84.88	87.25	91.00
F-Measure	90.13	98.38	82.35	88.07	94.76	93.36	87.05	88.24	80.71
Specificity	98.05	99.72	98.13	99.07	99.75	99.39	98.61	98.55	95.37

**Table A5:** Values of precision, recall, F-Measure, specificity of LOSO model SVM IWT (all values are expressed in %).

	HA	GL	FK	SP	CP	PH	TB	HB	HD
Precision	93.24	97.08	86.00	95.90	99.74	99.75	98.76	84.13	72.47
Recall	98.25	99.75	80.63	84.75	94.38	98.25	89.88	90.13	85.88
F-Measure	95.68	98.40	83.23	89.98	96.98	98.99	94.11	87.02	78.60
Specificity	99.02	99.59	98.26	99.51	99.97	99.97	99.85	97.73	95.76
